# Altered within- and between-host transmission under coinfection underpin parasite co-occurrence patterns in the wild

**DOI:** 10.1007/s10682-022-10182-9

**Published:** 2022-05-19

**Authors:** Suvi Sallinen, Hanna Susi, Fletcher Halliday, Anna-Liisa Laine

**Affiliations:** 1 Organismal and Evolutionary Biology Research Programme, University of Helsinki, Viikinkaari 1 (PO box 65), 00014 Helsinki, Finland; 2 Department of Evolutionary Biology and Environmental Studies, University of Zürich, CH-8057 Zurich, Switzerland

**Keywords:** *Phomopsis subordinaria*, *Plantago lanceolata*, Parasite community, Transmission, Virulence, Parasite-parasite interaction, Cross-kingdom interactions, Plant viruses

## Abstract

Interactions among parasite species coinfecting the same host individual can have far reaching consequences for parasite ecology and evolution. How these within-host interactions affect epidemics may depend on two non-exclusive mechanisms: parasite growth and reproduction within hosts, and parasite transmission between hosts. Yet, how these two mechanisms operate under coinfection, and how sensitive they are to the composition of the coinfecting parasite community, remains poorly understood. Here, we test the hypothesis that the relationship between within- and between-host transmission of the fungal pathogen, *Phomopsis subordinaria,* is affected by co-occurring parasites infecting the host plant, *Plantago lanceolata*. We conducted a field experiment manipulating the parasite community of transmission source plants, then tracked *P. subordinaria* within-host transmission, as well as between-host transmission to naïve recipient plants. We find that coinfection with the powdery mildew pathogen, *Podosphaera plantaginis*, causes increased between-host transmission of *P. subordinaria* by affecting the number of infected flower stalks in the source plants, resulting from altered auto-infection. In contrast, coinfection with viruses did not have an effect on either within- or between-host transmission. We then analyzed data on the occurrence of *P. subordinaria* in 2018 and the powdery mildew in a multi-year survey data set from natural host populations to test whether the positive association predicted by our experimental results is evident in field epidemiological data. Consistent with our experimental findings, we observed a positive association in the occurrence of *P. subordinaria* and historical powdery mildew persistence. Jointly, our experimental and epidemiological results suggest that within- and between-host transmission of *P. subordinaria* depends on the identity of coinfecting parasites, with potentially far-reaching effects on disease dynamics and parasite co-occurrence patterns in wild populations.

**Supplementary Information:**

The online version contains supplementary material available at 10.1007/s10682-022-10182-9.

## Introduction

Communities of parasite species infecting the same host individual (a phenomenon known as coinfection) (Cox 2001; Seabloom et al. 2009; Telfer et al. 2010), can profoundly alter the ecology and evolution of hosts and parasites (Lawn et al. 2006; Alizon et al. 2013; Bose et al. 2016), including the magnitude of parasite epidemics (Susi et al. 2015a; Halliday et al. 2017; Clay et al. 2020). Yet, our understanding of how a within-host parasite community simultaneously alters both within- and between-host parasite spread remains limited. Parasite spread often consists of growth and replication within an infected host, followed by transmission and colonization of new hosts, and both stages can be affected by the within-host parasite community (Bassanezi et al. 1998; Wintermantel et al. 2008; Tollenaere et al. 2016; Halliday et al. 2017). Research linking processes on these two scales under coinfection has recently advanced through the development of nested-models and implementation of the metacommunity framework (Mideo et al. 2008; Mihaljevic 2012; Richgels et al. 2013; Borer et al. 2016; Strauss et al. 2019). However, theory has outpaced empirical insights on how the composition of the within-host parasite community influences within- and between-host transmission, and how these dynamics underpin parasite coexistence patterns in natural populations.

A vast body of research has explored theoretical predictions and provided empirical evidence illustrating that disease severity and parasite growth and reproduction may differ under coinfection compared to single infections.(Taylor et al. 2002; Wintermantel et al. 2008; Mideo 2009; Alizon et al. 2013; Laine and Mäkinen 2018; Acevedo et al. 2019; Clerc et al. 2019). In empirical studies, coinfection often increases disease severity (Mukasa et al. 2006; Jolles et al. 2008; Laine and Mäkinen 2018; Kumar et al. 2020; Desai et al. 2021), and can accelerate auto-infection (López-Villavicencio et al. 2011; Susi et al. 2015b), whereby within-host replication is achieved via repeated transmission events from infected sites within that same host individual (Robinson 1976). Coinfection may also hinder growth and reproduction of some of the coinfecting parasites depending on the interaction mode (Bassanezi et al. 1998; Pedersen and Fenton 2007; Syller 2012; Bose et al. 2016; Desai et al. 2021). The type of interactions between the parasites (e.g. resource competition or cross-immunity), as well as the number and relatedness of the parasites are expected to alter parasite reproduction and disease severity (López-Villavicencio et al. 2007, 2011; Mideo et al. 2008; Alizon et al. 2013; Bose et al. 2016; Halliday et al. 2018, 2020). Empirically, it has been demonstrated that both the production of transmission propagules within a coinfected host and transmission between hosts may be affected by coinfection (Bassanezi et al. 1998; De Roode et al. 2005; Susi et al. 2015b; Halliday et al. 2017), and the effects on these two processes may be contradictory (Halliday et al. 2017). However, experiments that measure parasites on both of these scales are scarce and limited in the diversity of the parasite community considered. In particular, although cross-kingdom parasite coinfections are expected to be common, little is known about how these interactions affect transmission under natural conditions (Telfer et al. 2010; Auld et al. 2017; Clay et al. 2020).

Auto-infection can be a strong driver of disease occurrence and between-host transmission (Willocquet and Savary 2004; Vereijssen et al. 2007; Mundt 2009). High rates of auto-infection are expected to lead to higher transmission rates between individuals due to increased abundance of transmitting propagules, such as spores (Willocquet and Savary 2004). However, the number of transmission propagules within a host does not necessarily predict the rate of successful between-host transmission because transmission is a multi-faceted process affected by both the host population and environmental conditions, as well as the genotype of the host individual, the parasite strain, and coinfection of strains (Taylor et al. 1997; Antolin 2008; Tack et al. 2014; Susi et al. 2015b; McCallum et al. 2017). Indeed, theory predicts that the link between the within-host and between-host processes may be sensitive to whether or not a host is coinfected (Alizon et al. 2013). While examples of experimental work on parasite establishment and replication in diverse parasite-communities are found (Halliday et al. 2017; Clerc et al. 2019; Kumar et al. 2020), experimental work explicitly testing whether cross-kingdom parasite-parasite interactions change the dynamics of auto-infection and between-host transmission are rare.

How interactions among coinfecting parasites affect within- and between-host transmission may in turn determine which parasite species occur or co-occur in a given place and time. In natural host populations, non-random co-occurrences between parasite species are frequently observed (Seabloom et al. 2009; Telfer et al. 2010; Johnson and Buller 2011; Richgels et al. 2013; Rodelo-Urrego et al. 2013; Bolnick et al. 2020), and the mechanisms underpinning this phenomenon are under active research. Co-occurrence patterns result from shared responses to environmental variation among parasite species (Richgels et al. 2013; Lacroix et al. 2014; Borer et al. 2016; Tollenaere et al. 2016), are influenced by host genetic variation (Susi et al. 2015a; Audette et al. 2020; Sallinen et al. 2020), or represent the outcome of parasite-parasite interactions (Jolles et al. 2008; Telfer et al. 2010; Clay et al. 2019a). Parasite-parasite interactions can facilitate co-occurrence through multiple mechanisms, including both antagonistic and facilitative interactions among parasites (Bruno et al. 2003; Clay et al. 2019b, a). The infection dynamics of one parasite species could therefore scale-up to affect seasonal epidemics of another parasite, if coinfection affects the transmission of the parasites within and between hosts. While controlled experimental work has provided support for facilitative interactions among parasites (López-Villavicencio et al. 2011; Susi et al. 2015a; Kumar et al. 2020), the degree to which a change in within- and between-host dynamics in response to coinfection could scale up over time to promote coexistence in nature remains largely unknown.

Here, we used the fungal plant parasite, *Phomopsis subordinaria,* to test the hypothesis that the relationship between auto-infection and between-host transmission is affected by co-occurring parasites. We first performed a field experiment, where we manipulated the parasite community in the transmission source, and then allowed *P. subordinaria* to freely transmit to naïve recipient plants. Treatments consisted of *P. subordinaria* infecting a host alone and in seven different combinations of the powdery mildew *Podosphaera plantaginis*, *Plantago lanceolata latent virus*, and a virus bulk inoculum collected from wild *P. lanceolata* populations. We analyzed whether the severity of *P. subordinaria* infection varied among source plants in different treatments, and whether the parasite treatments had an effect on *P. subordinaria* transmission, measured as recipient plant infections. While such experiments provide powerful tests of potential mechanisms connecting coinfections to parasite transmission, the degree to which these results occur in nature is often unknown. Thus, we analyzed epidemiological data collected from wild host populations to test a hypothesis formulated from our experimental results. We hypothesize that a facilitative interaction could lead to a positive feedback between the powdery mildew and *P. subordinaria* in host populations. If this hypothesis was supported by the epidemiological data, we would find that *P. subordinaria* is more often present, and the populations are larger, in host populations with a history of powdery mildew infections.

Our results show that, of the coinfecting parasites, only the powdery mildew had a significant effect on *P. subordinaria,* leading to increased between-host transmission in the field experiment. This increased transmission was associated with higher virulence as the number of infected flower stalks as well as total number of flower stalks declined in the powdery mildew treatment over the course of the experiment indicating stalk mortality*.* Our analysis of field epidemiological data supported our hypothesis that this effect could lead to a positive feedback between these species over time, as we observed a positive association between *P. subordinaria* and the powdery mildew in wild host populations. Jointly our results show, for the first time, that interactions among coinfecting parasites can drive the development of parasite epidemics by altering within-host transmission with implications for parasite co-occurrence patterns and epidemiological dynamics in natural populations over time.

## Methods

### *Plantago lanceolata* parasite community in the Åland islands

*Plantago lanceolata* is a perennial herb (Sagar and Harper 1964) that occupies small, fragmented meadows forming a network of ~ 4000 populations in the Åland Islands (Ojanen et al. 2013). These populations have been monitored for size and location since the early 1990’s (Ojanen et al. 2013). *Plantago lanceolata* is self-incompatible and reproduces via seeds that drop close to the maternal plant, as well as vegetatively via side rosettes (Sagar and Harper 1964). In the Åland Islands (Finland), symptoms caused by two fungal parasites are commonly observed in *P. lanceolata* populations. *Phomopsis subordinaria* (Desm.) Trav. (telemorph *Diaporthe adunca* (Rob.) Niessl.) is an Ascomycota fungal pathogen usually found infecting *P. lanceolata* (de Nooij and van der Aa 1987). Powdery mildew *Podosphaera plantaginis* is an obligatory specialist Ascomycota fungus in the order Erysiphales. Virus-like symptoms are also frequently observed in *P. lanceolata* populations, and small RNA deep sequencing revealed numerous viruses infecting *P. lanceolata* in the Åland Islands (Susi et al. 2019; Norberg et al. 2021). *Plantago lanceolata latent virus* (PlLV) (Genus Capulaviridae) is among the five most common of these viruses, and PCR primers for detection of these five viruses have been designed (Susi et al. 2019).

*Phomopsis subordinaria* is transmitted via splash-dispersal but it needs a wound to enter the host plant (de Nooij and van der Aa 1987; Linders et al. 1996). Infection typically starts from below the ear of the flower (de Nooij and van der Aa 1987). Affected cells break, the tissue dies, and spore structures develop on the dead stalk tissue (de Nooij and van der Aa 1987) in 1–2 weeks (Susi et al. 2022). A visible discoloration of the necrotized tissue appears after a few days, and gradually proceeds down the stalk towards the rosette and into the leaves, often killing the host (de Nooij and van der Aa 1987). Flower stalks that are not infected, may not show any symptoms before the host dies (de Nooij and van der Aa 1987). Hosts are usually susceptible for infection, but the rate of fungal growth may differ among genotypes (de Nooij and van der Aa 1987). There is no previous research on the interactions of *P. subordinaria* and the other parasites used in this experiment. The host range of *P. subordinaria* is restricted, and it is usually not found in other plant species in nature, although experimental inoculation of other *Plantago* species has succeeded (de Nooij and van der Aa 1987). In the Åland Islands, *Phomopsis subordinaria* epidemics are typically restricted to a small fraction of plants within host populations with only up to 10% of plants affected (Laine 2003).

Annually up to 20% of *P. lanceolata* populations in the Åland Islands are host to powdery mildew *P. plantaginis*. The spores of *P. plantaginis* are wind-dispersed, but they typically travel short distances with most spores being deposited in immediate proximity of the infection source (Ovaskainen and Laine 2006; Tack et al. 2014). *Podosphaera plantaginis* persists in the fragmented host population network as a metapopulation through extinction and colonization events (Jousimo et al. 2014). There is a fitness cost of infection at the individual plant level (Susi and Laine 2015) and infection can reduce *P. lanceolata* population growth (Laine 2004; Penczykowski et al. 2014) but generally powdery mildews are not considered to cause significant host mortality in non-stressful environments (Bushnell 2002). The interaction between *P. plantaginis* and *P. lanceolata* is strain-specific (Laine 2004).

Virus infected plants are found in most surveyed populations, and viruses occur in varying frequencies both as single infections and as co-infections (Susi et al. 2019; Norberg et al. 2021). *Plantago lanceolata latent virus* (PlLV; Genus Capulaviridae) was first characterized from *P. lanceolata* in the Åland Islands (Susi et al. 2017), and occurs in low frequencies in these populations (Susi et al. 2019). No other known host species have been reported for PlLV. Infected plants range from asymptomatic to those displaying conspicuous yellowing of leaves (Susi et al. 2019). *Plantago lanceolata* genotypes vary in their likelihood to host this virus, which may be due to variation in susceptibility or variation in vector preference for certain genotypes (Sallinen et al. 2020). The aphid *Dysaphis plantaginea* has been identified as a potential vector in a laboratory transmission experiment (Susi et al. 2019), but the distribution and vector dynamics of PlLV and other viruses have not been studied.

### Experiment measuring the effect of parasite community on *P. subordinaria* transmission

**Fig. 1:**
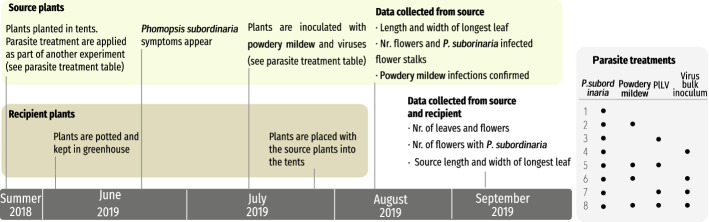
Timeline of the experiment testing transmission from varied within-host parasite communities to naïve recipient plants. Actions related to the transmission source plants, planted in a common garden inside insect web cages and inoculated with the parasite combinations shown on the gray table on the right, are shown on green. Actions related to healthy recipient plants, grown in a greenhouse and brought to the cages in pots, are listed on brown. Actions related to both recipient and source plants are listed on white. Lines link the actions to the timing on the timeline on dark gray. Gray table illustrates the different treatment combinations. All treatments had *Phomopsis subordinaria* and treatment 8 was a control with *P. subordinaria* only

To test whether *P. subordinaria* within-host and between-host transmission are affected by the co-occurring within-host parasite community, we conducted a two-month long (Fig. 1) field experiment in the summer of 2019 where source plants previously infected with *P. subordinaria* were inoculated with other parasites, and these source plants were surrounded with naïve recipient plants. The source plants represented eight different parasite treatments: 1. *P. subordinaria* alone (control), 2. *P. subordinaria* with powdery mildew, 3. *P. subordinaria* with PlLV, 4. *P. subordinaria* with bulk virus inoculum, 5. *P. subordinaria* with powdery mildew and PlLV, 6. *P. subordinaria* with powdery mildew and bulk virus inoculum, 7. *P. subordinaria* with PlLV and bulk virus inoculum, and 8. *P. subordinaria* with powdery mildew with both PlLV and bulk virus inoculum (Fig. 1). Within-host transmission was measured as change in the number of flower stalks infected by *P. subordinaria* in the beginning and the end of the experiment (Fig. 1), and transmission was measured as the established *P. subordinaria* infections on the recipient plants.

### Parasite material and inoculations

Inoculation of source plants with the powdery mildew (strain L10), were carried out by transferring spores on-site onto leaves of the source plants using a fine paintbrush. Spore material was grown on detached leaves on moist filter paper in Petri dishes in 16/8 h light–dark cycle and 21 degrees Celsius for two 14-day- cycles to produce enough material to inoculate each source plant with similar spore density and lesion size of 1 cm by 0.5 cm. Success of powdery mildew inoculation was checked in the first week of August and at the end of the experiment. Each source plant was checked for infection status (infected/not infected). The three source plant genotypes represent different powdery mildew resistance phenotypes, as has been observed during powdery mildew maintenance, and infection load varied among source plants.

In virus treatments, source plants were inoculated with viruses using sap extract from infected plants mixed with phosphate buffer. For PlLV inoculations, leaves of multiple PlLV infected plants, tested with specific PCR-primers, were crushed inside a plastic extraction bag (Bioreba, Switzerland) with phosphate buffer and stored in -20 °C until inoculation. The solution was melted pre-inoculation and plants were inoculated immediately by pressing 500 µl of the solution into the plant tissue with a syringe. For the virus bulk treatment, we collected leaves with typical virotic symptoms (yellow or red color, curliness) which are linked with higher likelihood of virus infection (Susi et al. 2019) from natural host populations in the Åland Islands. The leaves were stored in -80 °C until they were crushed inside an extraction bag (Bioreba, Switzerland) with phosphate buffer and pooled into one sample. The solution was immediately inoculated by pressing 500 µl of the solution into the plant tissue with a syringe. Plants in treatments with one type of virus inoculation were inoculated first to avoid contamination. Plants without virus treatments were mock-inoculated with buffer.

To confirm that the plants had received living virus particles in the inoculation, which triggers an immune response, we tested a subset of virus inoculated plants with specific PCR-primers. Because virus infections are difficult to identify based on symptoms (Prendeville et al. 2012; Susi et al. 2019) unlike *P. subordinaria* and the powdery mildew, we confirmed the inoculation success of virus-treated plants by testing a subset of 24 virus inoculated plants with specific PCR-primers (Susi et al. 2019; Sallinen et al. 2020). We tested eight source plants inoculated with PlLV only using PlLV specific primers. We also tested eight source plants inoculated with virus bulk inoculum and additional eight source plants inoculated with PlLV and virus bulk inoculum using four additional primers for detection of four additional viruses. These plants were selected so that both powdery mildew treated and powdery mildew free plants were included. The bulk virus inoculated plants may have received an even greater diversity of viruses, as the wild plants used for bulk virus inoculation may have been infected with viruses we cannot detect with these specific primers. For detailed methods of nuclease-extraction and PCR, and primer information, please see the Appendix S1.

### Experimental set-up

The source plants gained *P. subordinaria* infection prior to the transmission experiment, through inoculation in 2018 or through natural transmission from the inoculated plants during autumn and winter 2018. Neither the host plant nor *P. subordinaria* naturally exist in the area. The infection was caused by a single strain of *P. subordinaria* (ID: P23) collected from the Åland Islands. The additional parasite treatments with powdery mildew*,* PlLV, and bulk virus inoculum in this experiment correspond to treatments the plants received in the previous year (Fig. 1). The source plants represented three genotypes (ID:s 2220_m1, 511_11, 609_19) and were planted in sand inside 1 × 0.5 × 0.5 m insect-web-cages made out of plastic pest control fabric (Hortex, Finland) (Appendix S1: Fig. S1). In our transmission experiment, we used the generation of plants sprouting from the over-wintered rosettes as source plants. Six cages were used to replicate each of the eight parasite treatments, resulting in 48 cages, 18 source plants per treatment and 144 source plants in total. In each treatment, three of the six cages had been inoculated in 2018 and three had gained the infection through natural transmission from the inoculated plants. Different treatments were randomly located in a grid of cages with at least one-meter distance separating the cages. The cages with powdery mildew were located ten meters apart from the plants without powdery mildew, and separated with a plastic wall to obstruct any unlikely wind-dispersal. Six cages, each containing three healthy recipient plants, and no source infection (total 18 plants) were placed in the field among the cages as a negative control to confirm no transmission among the cages took place.

To measure *P. subordinaria* auto-infection in the source plants, we recorded the number of infected flower stalks in August at the beginning of the experiment, right after the recipient plants had been placed into the cages, and again at the end of September when the experiment ended (Fig. 1). To link the auto-infection results with overall host fitness, we counted the total number of flower stalks in the source plants in both time points. We calculated plant size by multiplying the number of leaves with leaf area of the longest leaf, using the equation: *A* = *πab*, where *a* is a half axis of the width of the longest leaf, and *b* is the half axis of the length of the longest leaf, and included this measurement in our analyses.

To measure between-host transmission of *P. subordinaria*, three month old greenhouse-grown “recipient” plants cloned from two maternal plant genotypes (ID:s 609_9 and 9021_7_ow) were placed within each cage. The recipient plants were kept in 10-by-10 cm pots with 30:70 sand-potting soil mixture. For each replicate cage, we placed three individuals representing each genotype. This resulted in six recipient plants in each cage, 36 plants per treatment, and 288 recipient plants in total. The recipients were placed into the cages two weeks after powdery mildew and virus inoculations were done in June and kept there until the end of the experiment at the end of September (Fig. 1). To measure transmission success, we recorded the infection status (infected/uninfected) of the recipient plants by checking the flower stalks for *P. subordinaria* symptoms.

### Statistical analysis of experimental results

All statistical analyses were conducted using R software (version 4.0.3) (R Core Team 2019). We first fit a linear mixed-effects model to test whether the parasite treatments affected *P. subordinaria* auto-infection rate in individual source plants. Change in the number of infected flower stalks during the experiments was used as the response variable. We used plant genotype (categorical, 3 levels), parasite treatment (categorical, 8 levels), plant size (continuous), and the origin of *P. subordinaria* infection as explanatory variables. The origin of *P. subordinaria* infection was included in the model as a categorical variable (two levels: inoculation in 2018 or transmission from the inoculated plants), as plants inoculated in 2018 had been infected for a longer time and hence, we expected them to be more heavily infected which may affect transmission. We included the cage as a random effect. We fit this model using lmer-function (package “lme4” version 1.1.25, Bates et al. 2015) and confirmed meeting the model assumptions using residual diagnostics. To determine the significance of the fixed effects, we calculated a likelihood-ratio test (function “Anova”, package “car”, Forx and Sanford 2019). To test which treatments differed from the control (*P. subordinaria* alone), we performed a pairwise comparison of the estimated marginal means and used Bonferroni p-value adjustment (functions “contrasts” and “emmeans”, package “emmeans” version 1.5.2.1, Lenth 2020). We only included source plants that flowered, because *P. subordinaria* infections begin in the flower stalks (de Nooij and van der Aa 1987; Laine 2003). We removed plants that did not get infected by *P. subordinaria* by the end of the experiment. Hence, 21 source plants were removed and 123 source plants were included in the analysis.

Next, we fit another linear mixed-effects model to analyze whether the change in total number of flower stalks in the source plants also varied among treatments. We calculated the change in total number of flower stalks (both diseased and healthy combined) from the beginning of the experiment to the end of the experiment. We analyzed the data with the same method, model structure, and the same post-hoc test as described above for auto-infection analysis. The only difference was the response variable.

Some of the powdery mildew inoculated plants in the experiment did not develop visible powdery mildew symptoms. To test the robustness of our modeling and results regarding the inoculated but not symptomatic plants, we also ran these two models by excluding the source plants that were inoculated with the powdery mildew but were not symptomatic. In addition, to test whether within the powdery mildew inoculated plants, the change in the number of infected flower stalks and the change in the total number of flowers varies between symptomatic and asymptomatic plants, we ran two models using the powdery mildew inoculated plants only. In this model, we used the symptom status as a binary explanatory variable. Please see “Supplementary analysis and results” for details (Appendix A1).

To test whether the effect of parasite treatment on auto-infection in the source plant leads to altered between-host transmission, we next fit a path model of recipient plant infections within cages. We only included recipient plants that produced flower stalks, since *P. subordinaria* infections mainly begin from the flower stalk, and plants without flower stalks were hence considered non-susceptible. We pooled the data of the source plants per cage, as all recipients within a cage were subjected to the source comprising of the three source plants.

Specifically, we fit a fully-mediated path model in two stages. In the first stage, the effect of treatments on the number of *P. subordinaria* infected flower stalks in the source in September was analyzed. The number of infected flower stalks in September represents a proxy of transmission source inoculum resulting from the auto-infection process. Powdery mildew inoculation (binary), PlLV inoculation (binary), and bulk virus inoculation (binary) were included as explanatory variables without interactions since our model of source-infections indicated no interactions (please see Results). Average source plant size in a cage and the origin of *P. subordinaria* infection were included as explanatory variables. The second stage tested the hypothesis that the treatment effect on *P. subordinaria* infections of the recipient plants is mediated by the source plants. The infections of recipients were included as response variable, and the number of *P. subordinaria* infected flower stalks, already modelled in the first stage, was used as an explanatory variable. We also included the number of flower stalks on the recipient and recipient genotype (categorical, two levels) as predictor on this stage. We fit the model with “lavaan” (Rosseel 2012) and “lavaan.survey” (Oberski 2014) with *P. subordinaria* infection status set as an ordered factor, and observations clustered by cage.

### Epidemiological data of *P. subordinaria* and the powdery mildew

To link our experiment to natural parasite dynamics, we use current day epidemiological data on *P. subordinaria* and historical epidemiological data on the powdery mildew in the Åland Islands. The size and location of the host plant populations in the Åland Islands have been surveyed since 1993, and powdery mildew occurrence and population size has been recorded every autumn since 2001 (Laine and Hanski 2006; for details on the survey protocol, please see Ojanen et al.). Specifically, powdery mildew populations size is estimated on a categorical scale: (1) 1–10 infected plants, (2) 10–50 infected plants, (3) 50–100 infected plants, (4) 100–1000 infected plants, (5) > 1000 infected plants (Laine and Hanski 2006). We chose this measure for powdery mildew population size instead of proportion infected, as this measure better descries the system: infections are typically clustered on small areas within the host populations (Laine 2006; Eck et al. 2020).

In September 2018, 261 host populations were surveyed for *P. subordinaria.* These populations were selected so that they represent different areas of the host population network (Appendix S1: Fig. S2) but were random in terms of previous powdery mildew history. We collected data on *P. subordinaria* presence and absence, as well as population size, measured on the same scale as powdery mildew population size. In addition, host population size was recorded as coverage in square meters (Ojanen et al. 2013).

### Statistical analysis of *P. subordinaria* and powdery mildew epidemiological data

Next, we tested whether we could detect a signal of our experimental results in wild host and parasite populations. Specifically, our experiment suggested that powdery mildew infection could increase *P. subordinaria* transmission (please, see Results). We hypothesized that if this effect was epidemiologically relevant a positive feedback over time could increase the likelihood of *P. subordinaria* presence and population size in host populations with higher powdery mildew persistence in the past. To test this hypothesis, we used *P. subordinaria* presence-absence and population size data collected in September 2018 and powdery mildew data from four years predating our *P. subordinaria* survey. For a robust estimate of powdery mildew persistence in the survey populations in the past, we calculated an average of powdery mildew population size (please see section “epidemiological data of *P. subordinaria* and powdery mildew for methods) over the years 2014–2017. In addition, our models included two other variables frequently observed to affect disease occurrence patterns: host population size in year 2018, measured as host plant coverage in square meters, and spatial connectivity. We included host population connectivity because previous research has demonstrated that it may affect parasite occurrence in spatially structured systems (Carlsson-Granér and Thrall 2015; Höckerstedt et al. 2018). Host population connectivity (*S*) for each population (*i*) was calculated as

$${S}_{i}=\sum \mathrm{exp}\left(-\alpha {d}_{ij}\right)\sqrt{{A}_{j}}$$

where *d*_*ij*_ is the Euclidian distance between patches *j* and *i* and *α* is the parameter of the negative exponential dispersal kernel, which was set to 1 km^−1^ (Jousimo et al. 2014). *A*_*j*_ is the area of habitat patch *j*. The square root transformation was used because it roughly corresponds to the scaling of host population size with patch area.

To test whether *P. subordinaria* occurrence in the survey populations is related to historical powdery mildew persistence, we fit a logistic regression model with *P. subordinaria* presence/absence as response variable and with three explanatory variables: average historical powdery mildew population size, connectivity of host population, and host plant coverage in 2018. To account for spatial dependency, the populations were divided into 11 spatial clusters (Appendix S1: Fig. S2) by hierarchical clustering with a 10 km cutoff value, the diameter from the centroid of a cluster and the cluster was used as a random effect in the model. Model performance was assessed with Tjur *R*^*2*^ coefficient of discrimination (Tjur 2009), which calculates the difference between the mean of the predicted probabilities of occurrence of *P. subordinaria* (presence vs. absence). The value is zero if the model predicts an equal number of success and failure, and hence is uninformative, and 1 if there is perfect separation.

To test whether *P. subordinaria* population size is related to historical powdery mildew persistence, we fit a cumulative link mixed effects model (CLMM, package “ordinal”, Christensen 2019) of *P. subordinaria* population size in 2018 with the same explanatory variables as for the presence/absence model. The significance of the fixed effects was determined with a likelihood ratio test comparing models with and without the given variable (function “anova”).

## Results

### Success of inoculations

To confirm that the source plants had been challenged by living parasite material, which triggers an immune response in the host and may lead to successful parasite establishment, we visually inspected the success of powdery mildew infection status of each source plants (infected/not infected) and checked virus infection status of 24 plants by using PCR detection. Powdery mildew was found on 36 of the 72 (50%) powdery mildew inoculated source plants. The prevalence varied among treatments with 11/18 (61%), 7/18 (39%), 8/18 (44%), and 10/18 (56%) plants included in powdery mildew only, powdery mildew with PlLV, powdery mildew with virus bulk inoculum, and powdery mildew with both PlLV and virus bulk inoculum treatment, respectively. In all but two cages, at least one source plant was infected. PlLV-infection was confirmed in 75% of PlLV-only inoculated plants (6/8). In plants inoculated with both PlLV and bulk inoculum, 88% of plants were confirmed PlLV-positive (7/8). Virus bulk inoculated plants (sixteen plants: eight with virus bulk only and eight with both PlLV and virus bulk) were tested for five viruses. Four viruses were detected from these plants: Betapartitivirus in 25%, Closterovirus in 88%, Caulimovirus in 88%, and PlLV in 88%, and Enamovirus in 0% of the tested plants. Coinfection was found in 89% (17 out of 18 plants) of bulk inoculated plants with 13% being host to two, 56% being host to three, and 25% being host to four viruses.

### *P. subordinaria* transmission experiment results

**Fig. 2:**
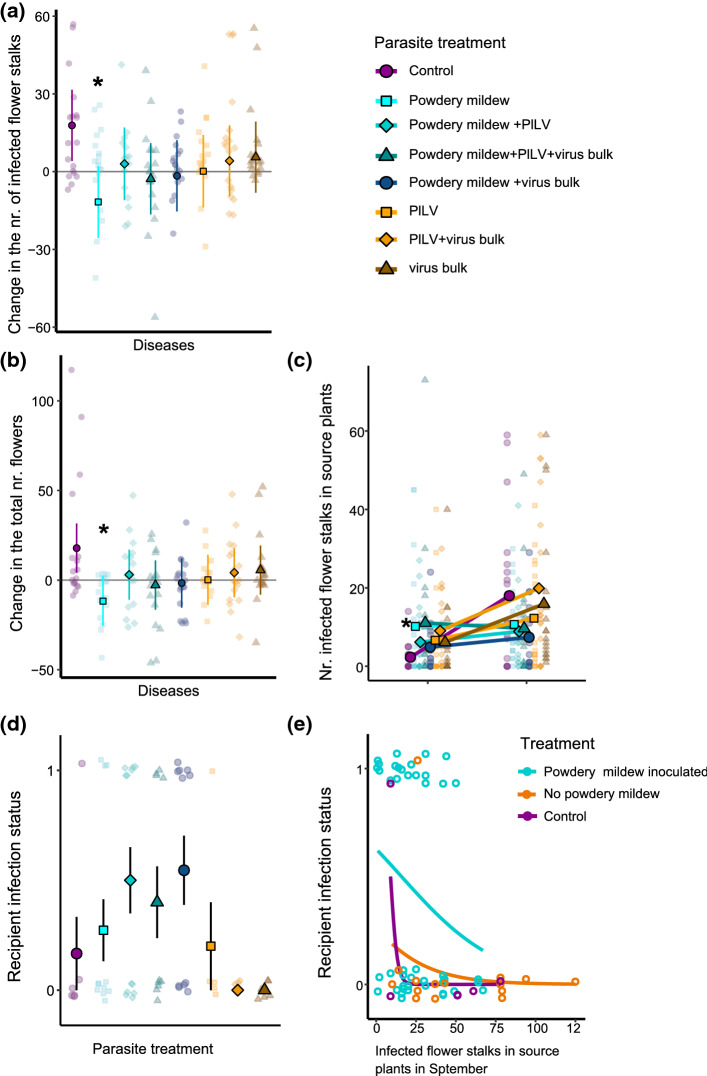
Results of the transmission experiment testing transmission from varied within-host parasite communities to naïve recipient plants shows that coinfection with powdery mildew causes more severe disease and increases transmission of *P. subordinaria*. **A** Change in the number of *Phomopsis subordinaria* infected flower stalks and **B** Total number of flower stalks from the beginning to the end of the experiment. Each unique color and shape combination shows a different parasite community treatment. Small points represent individual plants. Predicted averages and 95% confidence intervals calculated with the effects package in R are shown with large points and associated lines. An asterisk (*) shows the treatment where the change significantly differed from control in pairwise comparison. Treatments with powdery mildew are shaded with blue tones and treatments without it are shaded with yellow tones. Purple circle is the control with *P. subordinaria* only. **C** Number of *Phomopsis subordinaria* infected flower stalks in the source plants at the beginning and the end of the experiment. Each unique color and shape combination shows a different parasite community treatment. Each small point represents an individual plant. Large points show the average and a line shows the change of the average between the time points. An asterisk (*) shows the treatment where the change significantly differed from control in pairwise comparison (*p* < 0.05). Treatments with powdery mildew are shaded with blue tones and treatments without powdery mildew are shaded with yellow tones. The purple circle is the control with *P. subordinaria* only. **D** *Phomopsis subordinaria* infection status (infected/not infected) of recipient plants in different treatments in the end of the experiment. Each unique color and shape combination shows a different treatment. Treatments with powdery mildew are shaded with blue tones and treatments without it are shaded with yellow tones. Purple circle is the control with *P. subordinaria* only. Each small point represents one recipient plant and large points show the averages. Black lines show standard errors of the averages. **E** Relationship between the number of infected flower stalks in source plants in a cage at the end of the experiment on X-axis and recipient *P. subordinaria* infection status in Y-axis. Each empty circle represents an individual recipient plant. The lines show smoothed conditional means

Our first linear mixed-effects model of auto-infection (Appendix S1, Table S3) and likelihood-ratio test showed that the change in the number of *P. subordinaria* infected flower stalks from August to September differed between treatments (Fig. 2A, likelihood-ratio test χ^2^ = 20.084, df = 7, *p* < 0.01). It was also positively associated with plant size (χ^2^ = 43.916, df = 1, *p* < 0.01, Table S3). Plant genotype had no significant effect (χ^2^ = 4.535, df = 2, *p* = 0.1). As expected, the origin of *P. subordinaria* infection, whether the plant was inoculated or gained *P. subordinaria* through transmission from inoculated plants, significantly affected the disease progression (χ^2^ = 14.192, df = 1, *p* < 0.01). A post-hoc tests revealed that the change in the number of infected flower stalks declined under coinfection with the powdery mildew compared to the control of *P. subordinaria* only (estimate = -18.80, t-ratio = -3.746, df = 37.7, *p* < 0.01), but not compared to the other treatments (Fig. 2A, Appendix S1: Table S4). These results were robust regardless of whether we included all powdery mildew inoculated plants or only the subset that had powdery mildew symptoms, as shown in our supplementary analysis (Appendix: “Supplementary analysis and results”).

Our second linear-mixed effects model testing whether there was an effect on overall fitness showed that the change in the total number of flowers also declined in the powdery mildew inoculated plants compared to the control. Although a likelihood-ratio test of the significance of the main effects did not indicate a significant main effect of treatment with this model (χ^2^ = 10.3457, df = 7, *p* = 0.17, Appendix S1: Table S5), we conducted the post-hoc test and found that the change in the number of flower stalks significantly declined in powdery mildew treated source plants compared to the control (estimate = – 29.6, t-ratio = – 3.012, df = 38.3, p = 0.0321), but not compared to the other treatments (Fig. 2B, Appendix S1: Table S6). Plant size (*p* = 0.003, df = 1, χ^2^ = 8.5502) significantly affected the change in the total number of flowers while the origin of *P. subordinaria* infection (*p* = 0.76, df = 1, χ^2^ = 0.0927) and plant genotype (*p* = 0.2, df = 2, χ^2^ = 3.2125) did not. Our supplementary analysis that only included powdery mildew inoculated plants with visible symptoms produced similar results to the original model. However, in the post-hoc test, the significant difference between the powdery mildew treatment and healthy control shifted and was only marginally significant (estimate = – 3.0, t-ratio = 11.6 37.4, df = -2.836, *p* = 0.05).

**Fig. 3:**
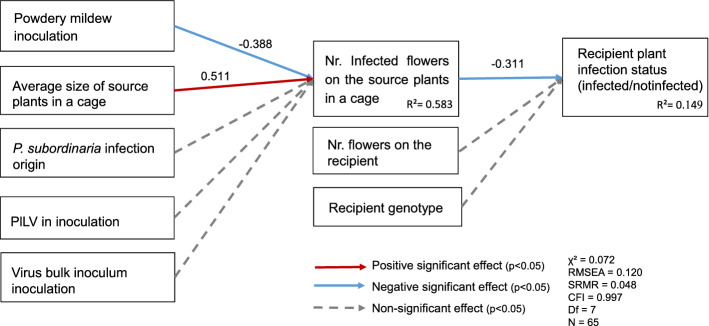
Results of a path model testing whether the effect of parasite community treatment on *Phomopsis subordinaria* transmission to recipient plants is caused by an effect of the treatment on the number of infected flower stalks in source plants. Coefficient values presented next to the significant paths are standardized estimates. For the full list of coefficients please see Table S8 in the Appendix S1

Next, we tested whether between-host transmission differed in the parasite treatments due to parasite treatment effect on the number of *P. subordinaria* infected flower stalks in the source plants by the end of the experiment. Out of 284 recipient plants included in the analysis, 65 produced flower stalks during the experiment and 21 plants (32.3%) became infected with *P. subordinaria* (Fig. 2D). The recipients produced 138 flower stalks in total and 42 flower stalks were infected (30.4%). We found signs of powdery mildew lesions on 29 out of the 144 (20%) powdery mildew inoculated recipient plants but only 6 out of the 44 plants (14%) that produced flower stalks and were therefore included in the model. Recipient plants in the control cages without source plants remained healthy during the experiment. To test whether between-host transmission differed among the parasite treatments due to parasite treatment effect on the number of infected flower stalks by the end of the experiment (a proxy for the transmission inoculum size that results from auto-infection) we fit a path model with two stages. In the first stage, treatment effects on source plant infections in September was tested with data pooled on cage level. In the first stage, the numbers of *P. subordinaria* infected flower stalks in the source plants were significantly lower with the powdery mildew (Fig. 3, Appendix S1: Table S8). This result is consistent with our auto-infection result indicating that the number of infected flower stalks declined from the beginning to the end of the experiment in the mildew alone treatment. In the second stage, the number of *P. subordinaria* infected flower stalks, in turn, negatively influenced the number of recipient plant infections, thereby generating an indirect positive relationship between the powdery mildew treatment and recipient infections that was mediated by the number of infected flower stalks on the source plants (Fig. 2E, Fig. 3, Appendix S1: Table S8). In other words, cages with powdery mildew treatment produced more infections on the recipient plants despite having fewer infected source flower stalks at the end of the experiment, because these cages also produced more infections per flower stalk (Fig. 2D, 2E, Fig. 3, Appendix S1: Table S8). Effects of both PlLV and bulk virus inoculation on the source plant infections and hence, on the recipient infections, were non-significant (Fig. 3, Appendix S1: Table S8).

### Association between *P. Subordinaria* and powdery mildew field epidemiological data

**Fig. 4:**
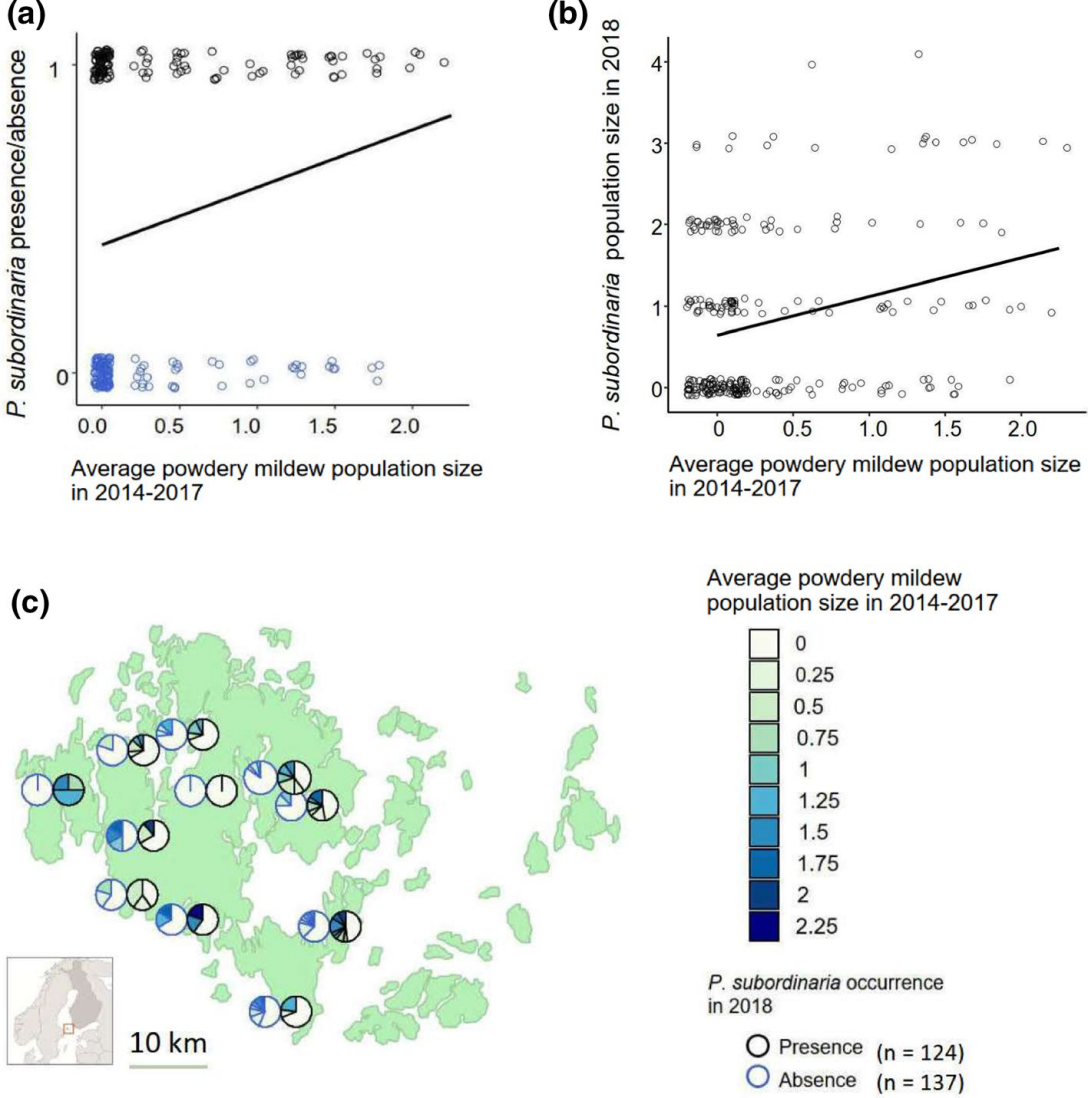
A positive relationship between *Phomopsis subordinaria* occurrence in 2018 and historical powdery mildew infections in 261 *Plantago lanceolata* populations in the Åland Islands. **A** Relationship between *P. subordinaria* presence (n = 124) and absence (n = 137) and average historical powdery mildew population size (calculated over years 2014–2017) in the surveyed host populations. Population size was measured on a categorical scale of five categories: (1) 1–10 infected plants, (2) 10–50 infected plants, (3) 50–100 infected plants, (4) 100–1000 infected plants, (5) > 1000 infected plants. The line denotes a smoothed average. **B** Relationship between *P. subordinaria* population size in 2018 and the average historical powdery mildew population size, both measured on the categorical scale described above. The line denotes a smoothed average. **C** Frequency of historical powdery mildew population size in the surveyed host populations grouped to 11 spatial clusters of 10–49 populations each. For the locations of individual populations and clusters, please see figure S2 in the Appendix S1

We found a positive association between *P. subordinaria* occurrence in 2018 and average historical powdery mildew population size across the 261 host populations (Appendix S1: Table S9). A likelihood-ratio test on the fixed effects suggested that *P. subordinaria* is more likely to occur in host populations with higher historical powdery mildew population size (Fig. 4, χ 2 = 7.0109, df = 1, *p* = 0.008) and higher host plant coverage (χ 2 = 5.0341, df = 1, *p* = 0.025, Fig. S3). Connectivity had no significant effect on *P. subordinaria* occurrence (χ 2 = 1.8808, df = 1, *p* = 0.17, Fig S3). Tjur’s *R*^2^ parameter of discrimination yielded a value of 0.13 indicating limited predictive power, which is not surprising given that ecological data sets are often inherently noisy and our model may lack some meaningful variables.

We then examined the effect of powdery mildew persistence in the populations on *P. Subordinaria* population sizes. Our cumulative link mixed effects model supports our hypotheses, as *P. Subordinaria* population size in 2018 is positively associated with historical powdery mildew persistence (Fig. 4B, Likelihood-Ratio statistic = 9.5912, df = 1, *p* = 0.002, Appendix S1: Table S10). Host plant coverage was positively correlated with *P. Subordinaria* population size (Likelihood-Ratio statistic = 26.229, df = 1, *p* < 0.001, Fig. S3) while host population connectivity had no effect (Likelihood-Ratio statistic = 0.102, df = 1, *p* = 0.745, Fig S3).

## Discussion

Although the unfolding of epidemics is strongly dependent on transmission dynamics, little attention has been paid to how auto-infection and between-host transmission are altered by multi-parasite settings, especially those that support cross-kingdom diversity. We combined data from a transmission experiment with epidemiological data from natural populations and find that coinfection of two fungal parasites can affect both within- and between-host transmission. We also found a link between these parasites in wild epidemiological data. The results support our hypothesis that the positive effect of the powdery mildew on *P. subordinaria* transmission observed in our experiment has scaled-up over time in natural host plant populations, where populations with more historical infections by one parasite also were more likely to host another parasite. Interestingly, coinfection by viruses commonly found in the natural populations had no effect on transmission of the focal parasite, *P. subordinaria* in the transmission experiment.

Our experimental results testing whether auto-infection and between-host transmission of *P. subordinaria* are affected by co-occurring parasites show that transmission between hosts increased when *P. subordinaria* is coinfecting the source plants with the powdery mildew*.* Between-host transmission is enhanced through an indirect positive effect, visualized as two negative paths in our path analysis: The number of infected flower stalks by the end of the experiment in the source plants –representing the transmission inoculum resulting from the within-host disease processes– is lower in the powdery mildew treatment. However, the more there are infected flower stalks, the less there is transmission. Hence, there appears to be a negative correlation between the number of infected flower stalks in the source plants and transmission under coinfection with the powdery mildew. As our source-plant analysis showed, the change in the number of infected flower stalks and the total number of flowers over time declines in the powdery mildew treated plants, indicating stalk mortality. *Phomopsis subordinaria* infection spreads in the flower stalk until the stalk is killed and the infection cannot be healed. Taken together, our results suggest that transmission to new hosts is higher when coinfection accelerates disease progression. This is probably because the dead stalks are likely to support more transmission propagules that the fungus produces after killing the plant tissue (de Nooij and van der Aa 1987). On the other hand, the increased transmission could also result from increased infectivity of the propagules under coinfection. However, the specific interaction between these two fungi is currently unknown. Our results are also consistent with previous research in foliar plant pathogens, where an increase in auto-infection is expected to increase between-host transmission, and the rare experimental examples have supported this prediction (Willocquet and Savary 2004; Susi et al. 2015a).

We did not observe an effect of the virus treatments on *P. subordinaria* transmission. Limited evidence suggests that virus infections reduce reproduction of fungal parasites in plants, although the negative consequences for the host may increase (Potter 1980; Omar et al. 1986; Marte et al. 1993; Bassanezi et al. 1998). It is also known that parasites from different kingdoms and with different feeding strategies trigger differential immune responses that further engage in cross-talk and are related to other stress-responses in plants (Glazebrook 2005; Pieterse et al. 2012; Collum and Culver 2016). Indeed, parasites may also interact via host immunity. Although we only confirmed virus infections in a subset of plants, the high prevalence of infections in these plants suggests that the inoculum has consisted of live virus particles. Hence, at the very least, the inoculation has challenged host immunity, which in some cases may have restricted the level of virus replication to such low levels that detection of the virus itself becomes difficult. Given that we do not quantify virus titer in the experimental plant after the inoculation, our experimental design may have most power to detect host immunity mediation of parasite-parasite interactions. How the various mechanisms play out under cross-kingdom parasite scenarios and affect parasite co-occurrence in the wild remains an important avenue for future research.

While common garden experiments provide powerful tests of potential mechanisms connecting coinfections to parasite transmission, the relevance of these findings in natural settings that are typically characterized by more stressful growing conditions and both biotic and abiotic complexity is often unknown (Johnson and Buller 2011; Clerc et al. 2019). We paired our experimental results with epidemiological data to test whether we could detect a signal of our experimental results in wild populations. We hypothesized that if the increased transmission under coinfection observed in our experiment was epidemiologically relevant, a positive feedback caused by this synergism over time would increases the likelihood of *P. subordinaria* presence and population size in host populations with more historical powdery mildew infections. Our analysis supported this hypothesis as we found a positive association between the occurrence and population sizes of these two parasites in wild host populations. This suggests that even weak interactions that can only be detected in experiments could have significant implications for how epidemics play out over time. Parasite-parasite interactions can promote co-existence in wild systems through multiple mechanisms (Clay et al. 2019a). Our results are suggestive of facilitation, which is expected to facilitate co-existence (Bruno et al. 2003), but it is also possible that other mechanisms, such as shared environmental responses, host density, or average host population resistance, contribute to the overall positive association in these wild populations. Our *P. subordinaria* data set only covers one growing season and investigating both the specific interaction between the parasites and temporal patterns of this parasite along with the rest of the parasite community in more detail could shed light on the multitude of mechanisms that lead to this positive association. Previous research in wild systems has shown that epidemics may be affected by coinfection (Telfer et al. 2010; Clay et al. 2020; Halliday et al. 2020), but no study to our knowledge has tested whether within-host dynamics can scale-up over time in wild populations, as we show here.

The notion that parasites and their hosts exist as communities is becoming a paradigm in parasite ecology, and parasite transmission is increasingly studied under a community context (Auld et al. 2017; Strauss et al. 2019). Our results contribute to growing evidence that coinfections affect the ecology and evolution of hosts and parasites and can be major drivers of epidemics (Lawn et al. 2006; Susi et al. 2015a). Effects of the within-host parasite community on replication and transmission remain key questions in parasite ecology because they are central parasite fitness measures, and hence, intimately linked with parasite evolution (Pedersen and Fenton 2007; Antolin 2008), as well as epidemiological dynamics (Lawn et al. 2006; Halliday et al. 2017; Clay et al. 2019b). Interactions among coinfecting parasites can drive the development of parasite epidemics by altering within-host processes with important implications for co-occurrence and possibly affect epidemics in natural populations over time through, for example, positive feedbacks.

## Supplementary Information

Below is the link to the electronic supplementary material.

Supplementary file1 (DOCX 654 kb)

Supplementary file2 (CSV 18 kb)

Supplementary file3 (CSV 34 kb)

## Data Availability

Upon acceptance of this manuscript, the data supporting the results will be made publicly available in Dryad repository.
