# Supplementary material for: Altered within- and between-host transmission under coinfection underpin parasite co-occurrence patterns in the wild
Source: Evol Ecol. 2022 May 19;37(1):131–51. doi: 10.1007/s10682-022-10182-9 (PMC9911512; doi:10.1007/s10682-022-10182-9)
Supplement: Supplementary file 1 — Supplementary file1 (DOCX 654 kb) [file 10682_2022_10182_MOESM1_ESM.docx]

*Supplementary material for*

Altered within- and between-host transmission under coinfection underpin parasite co-occurrence patterns in the wild

Authors: Sallinen Suvi, Susi Hanna, Halliday Feltcher, Laine Anna-Liisa

Journal: Evolutionary Ecology

Supplementary methods

**Virus detections of virus inoculated source plants**

We confirmed the success of virus inoculations by testing a subset of source plants using specific PCR-primers. We tested eight plants inoculated with PlLV only, eight plants inoculated with bulk virus inoculum, and eight plants inoculated with both. PlLV-only inoculated plants were tested for PlLV, and the sixteen plants inoculated with bulk virus inoculum were tested for four additional viruses occurring in the Åland Islands. Information about these viruses is listed in Table 1S below.

**Table 1S. Information of five viruses detected from the source plants.**

| Name in this manuscript | Species name | Family / Genus | Genome | Genome size | References |
| --- | --- | --- | --- | --- | --- |
| PlLV | *Plantago lanceolata latent virus* | Geminiviridae / Capulavirus | DNA, single-stranded | 2.8 kb | Susi et al. 2017 |
| Caulimovirus | *Plantago latent caulimovirus* | Caulimoviridae /Caulimovirus | DNA, double-stranded | 7.2-9.2 kb | Susi et al. 2019 |
| Betapartitivirus | *Plantago betapartitivirus* | Partitiviridae / Betapartitivurs | RNA, double stranded | 3-4.8 kb | Susi et al. 2019 |
| Enamovirus | *Plantago enamovirus* | Luteoviridae / Enamovirus | RNA, single stranded (+) | 5.6-6.0 kb | Susi et al. 2019 |
| Clolsterovirus | *Plantago closterovirus* | Closterviridae / Closterovirus | RNA, single-stranded (+) | 14.5-19.3 | Susi et al. 2019 |

**Nuclease-extractions of virus inoculated source plants**

In order to detect viruses with PCR, we first extracted DNA from PlLV inoculated plants, and DNA and RNA from bulk virus inoculated plants, as two of the viruses have a DNA-genome and three have an RNA-genome (Table S1). Nucleases were extracted from leaf samples collected from the source plants two weeks after virus inoculations, in the beginning of August. Leaf samples were collected from growing leaves of similar age using forceps that were sterilized with DNA-away between samples. Samples were immediately frozen in liquid nitrogen after detaching them from the plant and stored in -80 ºC.

DNA was extracted from 1 cm² of leaf tissue using CTAB-extraction (Lodhi et al. 1994). Briefly, leaf tissue was grinded into fine powder with liquid nitrogen and mixed with 500 µl of extraction buffer. Samples were incubated for 25 minutes in 60 ºC, after which 600 µl of cloroform-octanol (24:1) was mixed in followed by a phase-separation by centrifuging for 15 min in 6000 rpm in room temperature. The step was repeated twice. Next, the supernatant was extracted to a new tube and 1 ml of cold (-20 ºC) 95 % ethanol was added. The samples were precipitated in +4 ºC for 15-20 min. Samples were centrifuged for 3 minutes in 3000 rpm and additional 3 minutes in 6000 rpm. The ethanol was poured off and the pellet was washed with cool (+4 ºC) 70% ethanol. The remaining ethanol was evaporated by keeping the samples uncovered in +37 ºC for 30 minutes. DNA was diluted in 200 µl nuclease-free water. RNA was removed by adding 2 µl of RNase A and incubating in +37 ºC for 15 minutes. The samples were stored in -20.

RNA was extracted from 3 cm² of leaf tissue using a modified Chang et al. (1993) protocol with added phenol-cloroform extraction steps, using acid phenol (pH ~4). Leaf material was grinded into a fine powder in liquid nitrogen and immediately melted in +65 ºC isolation buffer. A volume of 800 µl of phenol-cloroform-IAA (25:24:1) was mixed in and samples were centrifuged for 15 minutes with 13 500 rpm in room temperature. The supernatant was extracted to a new tube and mixed with 800 µl of phenol-cloroform-IAA. Samples were centrifuged for 15 minutes with 13 500 rpm in room temperature. The supernatant was extracted to a new tube and mixed well with cloroform-IAA (24:1). Samples were centrifuged for 15 minutes with 13 500 rpm in room temperature. The supernatant was extracted to a new tube and mixed with 160 µl LiCl. Samples were left to precipitate over night in +4 ºC on ice. Next day, samples were centrifuged in +4 ºC for 30 minute in 10 000 rpm. Supernatant was removed and 500 µl of pre-warmed (+65 ºC) SSTE-buffer was added. Next, 1 ml of cloroform-IAA (24:1) was added and mixed well. Samples were centrifuged 15 minutes with 13 500 rpm in room temperature. The supernatant was extracted and mixed with 1 ml of cloroform-IAA (24:1). Samples were centrifuged for 15 minutes with 13 500 rpm in room temperature. The supernatant was extracted to a new tube and 920 µl of cold (-20 ºC) 95% ethanol was added. Following a two hour incubation in -20 ºC, the samples were centrifuged in +4 ºC for 20 minutes with 13 000 rpm. Ethanol was removed and the pellet was washed with cold (-20 ºC) 70% ethanol. Samples were centrifuged in +4 ºC for 20 minutes with 13 000 rpm. Ethanol was removed and remaining ethanol was precipitated on ice for 15 min. The RNA-pellet was resuspended in 25 µl of nuclease free water.

RNA was immediately translated into cDNA by reverse transcriptation by first mixing 2 ng of total RNA with 2 µl of Random Hexamer (50 µM, Invitrogen, USA) and nuclease free water to total volume of 17,125 µl. The samples were incubated in +70 ºC for 5 minutes. Next, 1 µl of Moloney Murine Leukemia Virus Reverse Transcriptase (M-MLV RT; Promega Corporation, USA), 0.625 μl of RiboLock RNaseinhibitor (Thermo Scientifi, USA), 5 μL M-MLV RT buffer (Promega Corporation, USA), and 1.25 μl of dNTP mix (10 mM, Thermo Scientifi, USA) were added. Total reaction volume was 37.41 μl. The samples were incubated in +37 ºC for 60 minutes and the finished cDNA was stored in -20.

**PCR-detection of viruses from virus inoculated source plants**

To detect a virus from a sample with PCR, 1 µl or DNA or cDNA was mixed with 5 µl of GoTaq Green 2X Master mix (Promega Corporation, USA), 1 µl of forward primer (100uM, 1:20 diluted in water 1:20), and 1 µl of reverse primer (100uM, diluted in water 1:20). In addition, 2 µl of water was added for a total reaction volume of 10 µl. PCR-reaction protocol included a 2 minute initial denaturation in 95 °C, followed by 35 cycles with the following steps: 40 seconds in 95 °C, 40 seconds annealing in 53–60 °C (please see Table S2 for primer specific temperatures), and 1 minute extension in 72 °C. The protocol ended in a final extension step of 72 °C for 5 minutes. PCR products were checked in 1.2 % agarose gel stained with ethidium bromide and the agarose gel was visualized with Gel Doc XR System (Bio-Rad Laboratories, Inc., USA).

**Table S2. Details of virus detection primers.** In “Primers”-column, “F” refers to forward primer and “R” to reverse primer

| Virus | Primers | Annealing temperature | Product size | Reference |
| --- | --- | --- | --- | --- |
| PlLV | F: GTGTTTAACAATGAAGTGAGCC  R: AATCCATCCACACATCCAATC | 60 | 117 nt | Sallinen et al. 2020 |
| Caulimovirus | F: AGGAGATGCCCATACTTTACC  R: GACTTGCCAGAACCTGATTTAC | 60 | 100 nt | Sallinen et al. 2020 |
| Betapartitivirus | F: TCCGTCCTGTTTATGCTGTTGA  R: TCTTGCAGACATAGTGTGAGGC | 53 | 974 nt | Susi et al. 2019 |
| Enamovirus | F: GGCTGGCCAAAGAAGGGG  R: GCCAGGTTAGTCGACGTGCTCT | 57 | 929 nt | Susi et al. 2019 |
| Closterovirus | F: GATTTACCCCAGAACTGTTGGGTG  R: CTAACTTCTTCAGTTAAAGCGCGAGAA | 50 | 790 nt | Susi et al. 2019 |

Supplementary tables

**Table S3. Model coefficients testing auto-infection in source plants.** Fixed effects of a linear mixed-effects regression model where change in the number of *Phomopsis subordinaria* infected flower stalks in source plants between the beginning and the end of the experiment is the response. For categorical variables, one level is a reference level included in the intercept. Significance of the main effects was determined using a likelihood-ratio test and is reported in the Results.

| Fixed effect | Estimate | Std.Error | t-value |
| --- | --- | --- | --- |
| Intercept | 5.894 | 4.101 | 1.437 |
| Powdery mildew | -18.797 | 5.017 | -3.747 |
| Powdery mildew + PlLV | -10.938 | 5.154 | -2.122 |
| Powdery mildew + PlLV + virus bulk inoculum | -11.439 | 5.064 | -2.259 |
| Powdery mildew + virus bulk inoculum | -9.396 | 5.037 | -1.865 |
| PlLV | -11.235 | 5.066 | -2.218 |
| PlLV + virus bulk inoculum | -2.360 | 5.015 | -0.471 |
| virus bulk | -4.837 | 5.011 | -0.965 |
| Genotype 511_11 | 3.043 | 2.975 | 1.023 |
| Genotype 609_19 | 5.862 | 2.775 | 2.112 |
| Area (scaled) | 8.601 | 1.298 | 6.627 |
| *P. subordinaria* origin (inoculation) | 9.683 | 2.570 | 3.767 |

**Table S4. Post-hoc test comparing the change in the number of *Phomopsis subordinaria* infected flower stalks in the source plants in the beginning and end of the experiment in August and September, respectively.** Results of pairwise comparison of the estimated marginal means (EMMs) calculated from the linear mixed-effects model presented in Table S3. P-values are Bonferroni adjusted for multiple comparisons. Comparisons between the control (*P. subordinaria* alone) denoted with “c” and each parasite treatment. This result is visualized in Figure 1a.

| Contrast | Estimate | SE | df | t.ratio | p-value |
| --- | --- | --- | --- | --- | --- |
| **c –** Powdery mildew | **-18.80** | **5.02** | **37.7** | **-3.746** | **0.0042** |
| c **–** PlLV | -11.24 | 5.07 | 38.9 | -2.217 | 0.2278 |
| c **–** bulk virus | -4.84 | 5.01 | 37.6 | -0.965 | 1.0000 |
| c **–** Powdery mildew + PlLV | -10.94 | 5.16 | 41.1 | -2.121 | 0.2803 |
| c **–** Powdery mildew + bulk virus | -9.40 | 5.04 | 38.1 | -1.865 | 0.4894 |
| c **–** PlLV + bulk virus | -2.36 | 5.02 | 37.6 | -0.471 | 1.0000 |
| c – *P. plantaginis* + PlLV + bulk virus | -11.44 | 5.07 | 38.7 | -2.258 | 0.2076 |

**Table S5. Fixed effects of a model testing how change in the total number of flower stalks changes depending on treatment.** Response variable is change in total number of flowers, including both *Phomopsis subordinaria* infected flowers and healthy flowers. For categorical variables, one level is a reference level included in the intercept. Significance of the main effects was determined using a likelihood-ratio test and is reported in the Results.

| Fixed effect | Estimate | Std.Error | t-value |
| --- | --- | --- | --- |
| Intercept | 13.245 | 7.620 | 1.738 |
| Powdery mildew | -29.573 | 9.819 | -3.012 |
| Powdery mildew + PlLV | -14.835 | 9.929 | -1.494 |
| Powdery mildew + PlLV + virus bulk inoculum | -20.623 | 9.860 | -2.092 |
| Powdery mildew + virus bulk inoculum | -19.488 | 9.837 | -1.981 |
| PlLV | -17.675 | 9.907 | -1.784 |
| PlLV + virus bulk inoculum | -13.694 | 9.817 | -1.395 |
| virus bulk | -12.283 | 9.813 | -1.252 |
| Genotype 511_11 | 6.323 | 3.576 | 1.768 |
| Genotype 609_19 | 4.305 | 3.333 | 1.292 |
| Area (scaled) | 4.917 | 1.682 | 2.924 |
| *P. subordinaria* origin (inoculation) | 1.513 | 4.967 | 0.305 |

**Table S6. Post-hoc test comparing the change in total number of flower stalks in the source plants.** Results of a pairwise comparison of the estimated marginal means (EMMs) calculated from the linear mixed-effects model presented in Table S5. Comparisons between the control (*P. subordinaria* alone) denoted with “c” and each parasite treatment. This result is visualized in Figure 1b.

| Contrast | Estimate | SE | df | t.ratio | p-value |
| --- | --- | --- | --- | --- | --- |
| **c – Powdery mildew** | **-29.6** | **9.82** | **38.3** | **-3.012** | **0.0321** |
| c **–** PlLV | -17.7 | 9.91 | 39.5 | -1.784 | 0.5749 |
| c **–** bulk virus | -12.3 | 9.81 | 38.2 | -1.252 | 1.0000 |
| c **–** Powdery mildew + PlLV | -14.8 | 9.93 | 39.9 | -1.494 | 1.0000 |
| c **–** Powdery mildew + bulk virus | -19.5 | 9.84 | 38.5 | -1.981 | 0.3833 |
| c **–** PlLV + bulk virus | -13.7 | 9.82 | 38.2 | -1.395 | 1.0000 |
| c – Powdery mildew + PlLV + bulk virus | -20.6 | 9.86 | 38.8 | -2.091 | 0.3016 |

**Table S8. Coefficients of a path-model testing whether the effect of the powdery mildew treatment on transmission to recipient plants is caused by an effect in the source plants.** Variable names with “infection” refer to *P. subordinaria* infections. Upper panel shows results of the model referred as “stage 2” and lower panel “stage 1” in the main text.

| Response | Coefficient | Estimate | Std.Err. | z-value | | P(>\|z\|) | | | Std.all | |
| --- | --- | --- | --- | --- | --- | --- | --- | --- | --- | --- |
| Recipient infection status (0/1) | Nr. flowers | 0.057 | 0.039 | | 1.455 | | 0.146 | 0.178 | |  |
|  | Genotype | -0.072 | -0.072 | | -0.583 | | 0.560 | -0.071 | |  |
|  | **Nr. source infected flowers** | **-0.006** | **0.001** | | **-4.154** | | **0.000** | **-0.311** | |  |
| Nr. source infected flowers (numerical) | **Powdery mildew treated (0/1)** | **-21.560** | **10.169** | | **-2.120** | | **0.034** | **-0.388** | |  |
|  | PlLV treated (0/1) | 10.925 | 10.925 | | 1.152 | | 0.249 | 0.211 | |  |
|  | Bulk virus treated (0/1) | 12.014 | 9.536 | | 1.260 | | 0.208 | 0.233 | |  |
|  | **Average size in the cage** | **0.006** | **0.003** | | **2.357** | | **0.018** | **0.511** | |  |
|  | Source infection acquisition method | 11.393 | 7.177 | | 1.587 | | 0.112 | 0.221 | |  |
|  |  |  |  | |  | |  |  | |  |

**Table S9. Coefficients of a logistic regression model of *P. subordinaria* presence and absence in 261 populations surveyed in 2018 in the Åland Islands.**

| Fixed effect | Estimate | Std.Error | z-value | p-value |  |
| --- | --- | --- | --- | --- | --- |
| Intercept | -0.784542 | 0.308231 | -2.545 | 0.0109 |  |
| **Mean powdery mildew abundance** | **0.689082** | **0.260246** | **2.648** | **0.0081** |  |
| ***Plantago lanceolata* coverage in 2018** | **0.085179** | **0.037964** | **2.244** | **0.0249** |  |
| *Plantgo lanceolata* population connectivity | 0.013090 | 0.009545 | 1.371 | 0.1702 |  |

**Table S10. Coefficients of a cumulative link mixed effects model of *P. subordinaria* population size in 261 populations surveyed in 2018 in the Åland Islands.**

| Fixed effect | Estimate | Std.Error | z-value | p-value |  |
| --- | --- | --- | --- | --- | --- |
| **Mean powdery mildew abundance** | **0.70689** | **0.22830** | **3.096** | **0.00196** |  |
| ***Plantago lanceolata* coverage in 2018** | **0.74399** | **0.16885** | **4.406** | **< 0.0001** |  |
| *Plantgo lanceolata* population connectivity | -0.05002 | 0.15682 | -0.319 | 0.74976 |  |

Supplementary figures

**Fig S1. Insect cages with inoculated source plants planted in sand and naïve recipient plants placed with them in pots.**


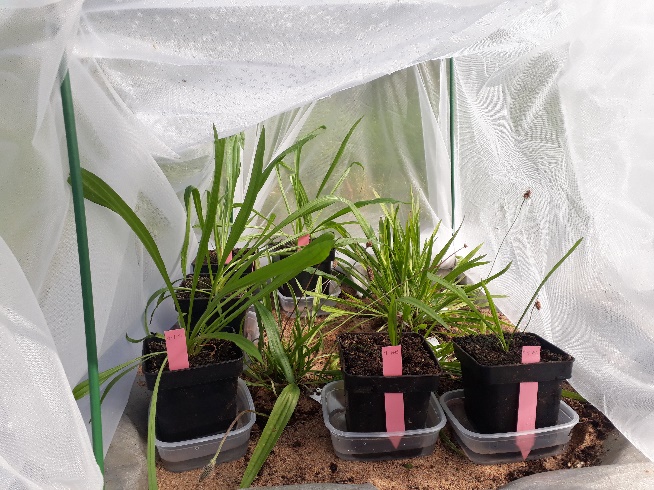

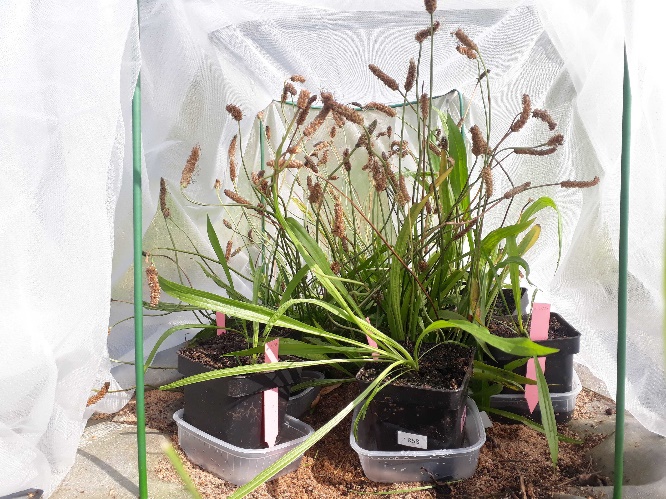


**Fig S2**. **Locations of 261 *Plantago lanceolata* populations where epidemiological data of *Phomopsis subordinaria* and powdery mildew was collected.** Populations were divided into spatial clusters using a hierarchical clustering and 10 kilometer distance cutoff (diameter from the centroid of a cluster). Each point is a population and colors denote the clusters.


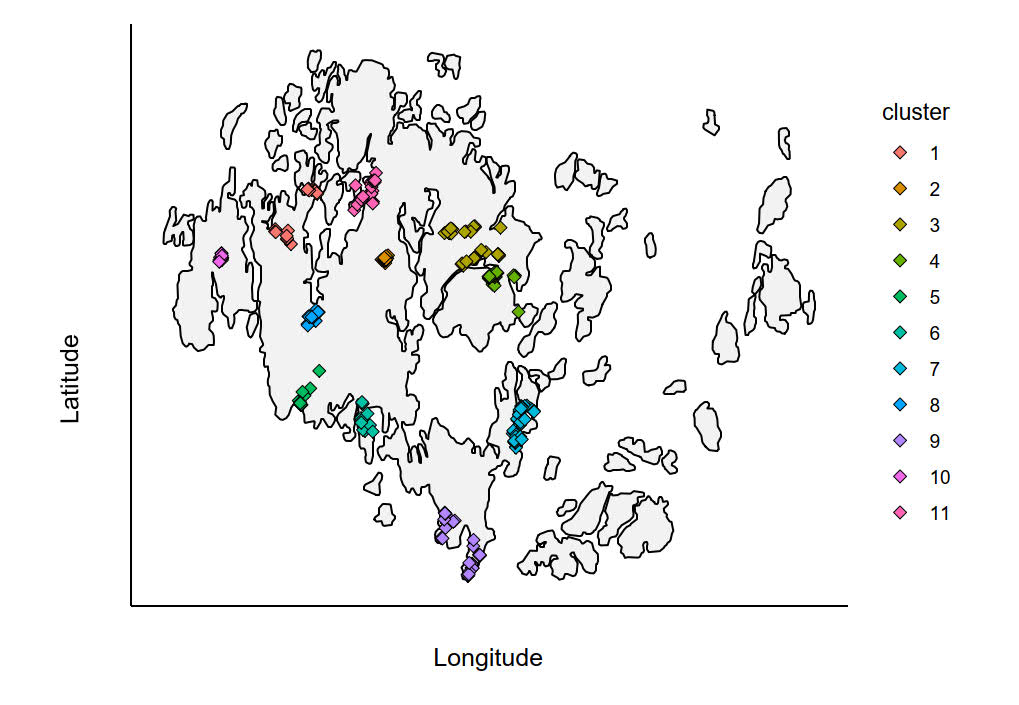


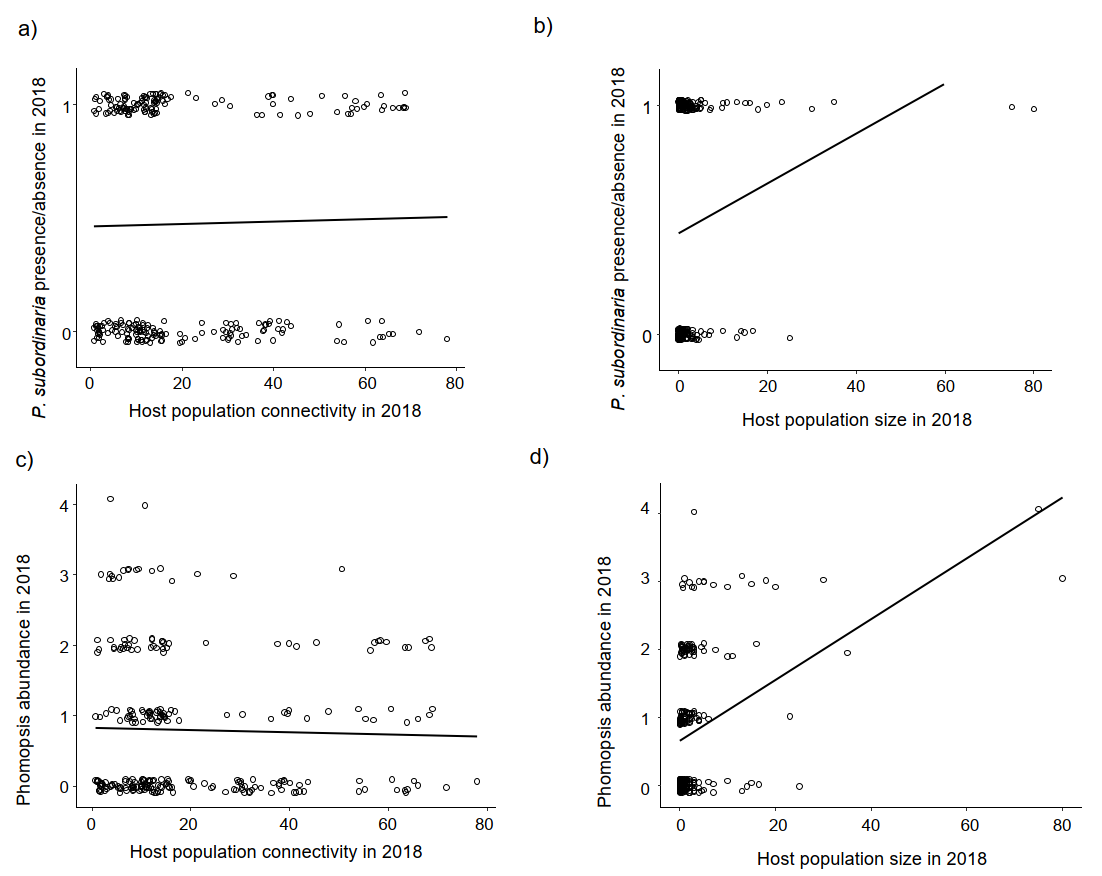
**Fig S3.** The relationship between host population connectivity and host population size, measured as host plant coverage in square meters, with *P. subordinaria* presence/absence in a) and b), and with *P. subordinaria* abundance in c) and d), measured on a categorical scale of five categories: 1) 1-10 infected plants, 2) 10-50 infected plants, 3) 50-100 infected plants, 4) 100-1000 infected plants, 5) >1000 infected plants.

**SUPPLEMENTARY ANALYSIS AND RESULTS**

**Analysis of powdery mildew symptomic infections in the field experiment**

To test how powdery mildew symptom status (visually detected infection/no symptoms detected) of the powdery mildew inoculated plants affects auto-infection, measured as the change in the number of *P. subordinaria* infected flower stalks, and the total number of flower stalks, we first ran two models with the same model structure as the original models but only included those powdery mildew inoculated plants that had visually confirmed infections. Both models included plant genotype (categorical, 3 levels), parasite treatment (categorical, 8 levels), plant size (continuous), and the origin of *P. subordinaria* infection (inoculated/transmitted) as explanatory variables. Cage was included as a random effect in both models. We fit these models using lmer-function (package “lme4” version 1.1.25, Bates et al. 2015) and determined the significance of the fixed effects by calculating a likelihood-ratio test (function “Anova”, package “car”, Forx and Sanford 2019). To test which treatments differed from the control (*P. subordinaria* alone), we performed a pairwise comparison of the estimated marginal means and used Bonferroni p-value adjustment (functions “contrasts” and “emmeans”, package “emmeans” version 1.5.2.1, Lenth 2020).

To further test whether powdery mildew symptomatic infections affected auto-infection and the total number of flower stalks, we ran two models with only the powdery mildew inoculated plants. These models included a binary explanatory variable for powdery mildew infection status (visually confirmed infection /no detected infection) with an interaction with the virus inoculation treatments PlLV (binary, yes/no) and bulk virus inoculum (binary, yes/no). The models also included plant genotype (categorical, 3 levels), parasite treatment (categorical, 8 levels), plant size (continuous), and the origin of *P. subordinaria* infection (inoculated/transmitted) as explanatory variables as fixed effects and cage was included as a random effect. We fit these models using lmer-function (package “lme4” version 1.1.25, Bates et al. 2015) and determined the significance of the fixed effects by calculating a likelihood-ratio test (function “Anova”, package “car”, Forx and Sanford 2019).

**Results of the powdery mildew symptom analysis**

Our first model testing whether auto-infection varies among source plants and only included those powdery mildew inoculated plants that had visually detected symptoms produced similar results as the original model including all source plants (Table S11, S12). Parasite inoculation treatment was significant (P = 0.006, df = 7, χ² = 19.7787) along with plant genotype (P = 0.02, df = 2, χ² = 7.4881), size (P < 0.001, df = 1, χ² = 41.0018), and origin of *P. subordinaria* infection (P < 0.001, df = 1, χ² = 12.2031). Post-hoc test comparing treatments to the control showed that the powdery mildew alone treatment differs form the healthy control similarly to the original model: the values were significantly lower (Table S12).

Our second model testing whether the total number of flower stalks also corresponds to the original model (Table S13, S14) showed that parasite inoculation treatment was not significant (P= 0.24, df=7, χ² =9.1662) and the post-hoc test comparing treatments shows that the previously significant difference between powdery mildew alone and the control (estimate = -29.6, t-ratio = -3.012, df = 38.3, P = 0.0321) became to marginally significant (estimate = -33.0, t-ratio = 11.6 37.4 , df = -2.836, P = 0.0514). There was not a significant effect of plant genotype (P = 0.549, df = 2, χ² = 1.1993), or origin of *P. subordinaria* infection (P = 0.734, χ² = 0.1134, df =1) and only plant size (P = 0.013, df = 1, 6.1511) was significant.

Our analysis of whether auto-infection (Table S15) and the change in the total number of flowers (Table S16) differed in powdery mildew inoculated plants depended on visual symptomatic infection showed that there was not a significant difference among plants with or without visually detected infection. In the model of auto-infection, there was no significant interaction between virus treatment and powdery mildew infection status (χ² = 0.2613, df = 3, P = 0.96,) nor significant main effects of virus treatment (χ² = 1.1648, df = 3, P = 0.76) or powdery mildew infection status (χ² = 0.2106, df = 1, P = 0.64630). Plant genotype (χ² = 4.0785, df = 2, P = 0.13012) and plant size (χ² = 3.4326, df = 1, P = 0.06392) were also non-significant, but the origin of *P. subordinaria* infection was significant (χ² = 6.4504, df = 1, P = 0.011). Also for the change in total number of flowers, there were no interaction between (χ² = 0.8240, df = 3, P = 0.8437), nor significant main effects of virus treatment (χ² = 3.4479, df = 3, P = 0.3276) or powdery mildew infection status (χ² = 0.0030, df = 1, P = 0.9564). Plant size (χ² =1.3718, df = 1, P = 0.2415), plant genotype (χ² = 3.3671, df =2, P =0.1857), and the origin of *P. subordinaria* infection (χ² = 3.3671, df = 2, P =0.1857)

Our results jointly suggest that the powdery mildew inoculation rather than symptomatic powdery mildew infection is driving the pattern we observe where powdery mildew affects *P. subordinaria* auto-infection by reducing the number of infected flower stalks over the course of experiment. For auto-infection, it does not matter whether we include both symptomic and non-symptomatic powdery mildew inoculated plants. For the total number of flower stalks, there is some difference only if we include all powdery mildew challenged plants indicating that the inoculation of powdery mildew may have an effect on it’s own. This is additionally supported by our other additional analysis showing that among powdery mildew inoculated plants, there is not a significant difference among plants that had or did not have visually detected infections.

**Table S11. Model coefficients testing auto-infection in source plants with only those powdery mildew inoculated plants included that had symptoms.** Fixed effects of a linear mixed-effects regression model where change in the number of *Phomopsis subordinaria* infected flower stalks in source plants between the beginning and the end of the experiment is the response. For categorical variables, one level is a reference level included in the intercept. Significance of the main effects was determined using a likelihood-ratio test and is reported in the Results.

| Fixed effect | Estimate | Std.Error | t-value |
| --- | --- | --- | --- |
| Intercept | 5.389 | 4.775 | 1.129 |
| Powdery mildew | -24.012 | 6.403 | -3.750 |
| Powdery mildew + PlLV | -12.720 | 7.116 | -1.787 |
| Powdery mildew + PlLV + virus bulk inoculum | -14.839 | 6.872 | -2.159 |
| Powdery mildew + virus bulk inoculum | -11.469 | 6.456 | -1.776 |
| PlLV | -10.948 | 5.734 | -1.909 |
| PlLV + virus bulk inoculum | -1.998 | 5.686 | -0.351 |
| virus bulk | -4.720 | 5.681 | -0.831 |
| Genotype 511_11 | -0.402 | 3.884 | -0.104 |
| Genotype 609_19 | 7.189 | 3.203 | 2.244 |
| Area (scaled) | 9.938 | 1.552 | 6.403 |
| *P. subordinaria* origin (inoculation) | 11.493 | 3.290 | 3.493 |

**Table S12. Post-hoc test comparing the change in the number of *Phomopsis subordinaria* infected flower stalks in the source plants in the beginning and end of the experiment in August and September, respectively and with only those powdery mildew inoculated plants included that had symptoms.** Results of pairwise comparison of the estimated marginal means (EMMs) calculated from the linear mixed-effects model presented in Table S3. P-values are Bonferroni adjusted for multiple comparisons. Comparisons between the control (*P. subordinaria* alone) denoted with “c” and each parasite treatment.

| Contrast | Estimate | SE | df | t.ratio | p-value |
| --- | --- | --- | --- | --- | --- |
| **c – Powdery mildew** | **-24.01** | **6.42** | **39.0** | **-3.740** | **0.0041** |
| c **–** PlLV | -10.95 | 5.74 | 27.0 | -1.908 | 0.4692 |
| c **–** bulk virus | -4.72 | 5.68 | 26.2 | -0.831 | 1.0000 |
| c **–** Powdery mildew + PlLV | -12.72 | 7.13 | 48.8 | -1.784 | 0.5648 |
| c **–** Powdery mildew + bulk virus | -11.47 | 6.48 | 39.7 | -1.770 | 0.5904 |
| c **–** PlLV + bulk virus | -2.00 | 5.69 | 26.3 | -0.351 | 1.0000 |
| c – Powdery mildew + PlLV + bulk virus | -14.84 | 6.88 | 43.2 | -2.155 | 0.2572 |

**Table S13 Fixed effects of a model testing how change in the total number of flower stalks changes depending on treatment but only including those powdery mildew inoculated plants that had symptoms.** Response variable is change the total number of flowers, including both *Phomopsis subordinaria* infected flowers and healthy flowers. For categorical variables, one level is a reference level included in the intercept. Significance of the main effects was determined using a likelihood-ratio test and is reported in the Results.

| Fixed effect | Estimate | Std.Error | t-value |
| --- | --- | --- | --- |
| Intercept | 14.112 | 8.697 | 1.623 |
| Powdery mildew | -32.991 | 11.624 | -2.838 |
| Powdery mildew + PlLV | -16.482 | 12.516 | -1.317 |
| Powdery mildew + PlLV + virus bulk inoculum | -19.637 | 12.313 | -1.595 |
| Powdery mildew + virus bulk inoculum | -22.497 | 11.675 | -1.927 |
| PlLV | -17.488 | 11.079 | -1.578 |
| PlLV + virus bulk inoculum | -13.671 | 10.989 | -1.244 |
| virus bulk | -12.281 | 10.984 | -1.118 |
| Genotype 511_11 | 2.733 | 4.841 | 0.565 |
| Genotype 609_19 | 4.465 | 4.080 | 1.094 |
| Area (scaled) | 5.017 | 2.023 | 2.480 |
| *P. subordinaria* origin (inoculation) | 2.020 | 5.999 | 0.337 |

**Table S14. Post-hoc test comparing the change in total number of flower stalks in the source plants but only including those powdery mildew inoculated plants that had symptoms.** Results of a pairwise comparison of the estimated marginal means (EMMs) calculated from the linear mixed-effects model presented in Table S5. Comparisons between the control (*P. subordinaria* alone) denoted with “c” and each parasite treatment.

| Contrast | Estimate | SE | df | t.ratio | p-value |
| --- | --- | --- | --- | --- | --- |
| **c – Powdery mildew** | **-33.0** | **11.6** | **37.4** | **-2.836** | **0.0514** |
| c **–** PlLV | -17.5 | 11.1 | 31.4 | -1.578 | 0.8718 |
| c **–** bulk virus | -12.3 | 11.0 | 30.4 | -1.118 | 1.0000 |
| c **–** Powdery mildew + PlLV | -16.5 | 12.5 | 41.4 | -1.316 | 1.0000 |
| c **–** Powdery mildew + bulk virus | -22.5 | 11.7 | 38.0 | -1.925 | 0.4321 |
| c **–** PlLV + bulk virus | -13.7 | 11.0 | 30.4 | -1.244 | 1.0000 |
| c – Powdery mildew + PlLV + bulk virus | -19.6 | 12.3 | 38.9 | -1.594 | 0.8339 |

**Table S15. Coefficients of a model testing how the change in the number of *P. subordinaria* infected flower stalks in the powdery mildew inoculated plants is affected by powdery mildew symptom status and virus treatments.** For categorical variables, one level is a reference level included in the intercept. Significance of the main effects was determined using a likelihood-ratio test and is reported in the Results.

| Fixed effect | | Estimate | | Std.Error | t-value |
| --- | --- | --- | --- | --- | --- |
| Intercept | | -11.3506 | | 6.9788 | -1.626 |
| Powdery mildew symptom (no) |  | | -3.1934 8.5051 -0.375 | | |
| PlLV | | 2.7028 | | 7.8004 | 0.347 |
| PlLV and virus bulk inoculum | | 1.9409 | | 7.7991 | 0.249 |
| virus bulk | | 6.4229 | | 8.0004 | 0.803 |
| Genotype 511_11 | | 7.9352 | | 5.3573 | 1.481 |
| Genotype 609_19 | | 7.9375 | | 4.4622 | 1.779 |
| Area (scaled) | | 4.8060 | | 2.5940 | 1.853 |
| *P. subordinaria* origin (inoculation) | | 10.2529 | | 4.0370 | 2.540 |
| Powdery mildew symptom (no) *PlLV | | 3.6395 | | 10.5248 | 0.346 |
| Powdery mildew symptom (no) *virus bulk | | -0.9938 | | 10.7103 | -0.093 |
| Powdery mildew symptom (no) *PlLV and virus bulk | | 2.3936 | | 9.9203 | 0.241 |
|  | |  | |  |  |

**Table S16. Coefficients of a model testing how the change in the total number of flowers in the powdery mildew inoculated plants is affected by powdery mildew symptom status and virus treatments.** For categorical variables, one level is a reference level included in the intercept. Significance of the main effects was determined using a likelihood-ratio test and is reported in the Results.

| Fixed effect | | Estimate | | Std.Error | t-value |
| --- | --- | --- | --- | --- | --- |
| Intercept | | -11.3506 | | 6.9788 | -1.626 |
| Powdery mildew symptom (no) |  | | -3.1934 8.5051 -0.375 | | |
| PlLV | | 2.7028 | | 7.8004 | 0.347 |
| PlLV and virus bulk inoculum | | 1.9409 | | 7.7991 | 0.249 |
| virus bulk | | 6.4229 | | 8.0004 | 0.803 |
| Genotype 511_11 | | 7.9352 | | 5.3573 | 1.481 |
| Genotype 609_19 | | 7.9375 | | 4.4622 | 1.779 |
| Area (scaled) | | 4.8060 | | 2.5940 | 1.853 |
| *P. subordinaria* origin (inoculation) | | 10.2529 | | 4.0370 | 2.540 |
| Powdery mildew symptom (no) *PlLV | | 3.6395 | | 10.5248 | 0.346 |
| Powdery mildew symptom (no) *virus bulk | | -0.9938 | | 10.7103 | -0.093 |
| Powdery mildew symptom (no) *PlLV and virus bulk | | 2.3936 | | 9.9203 | 0.241 |
|  | |  | |  |  |

Literature

Chang, S., J. Puryear, and J. Cairny. 1993. A simple and efficient method for isolating RNA from pine trees. Plant Molecular Biology Reporter 11:113–116.

Lodhi, M. A., G. N. Ye, N. F. Weeden, and B. I. Reisch. 1994. A simple and efficient method for DNA extraction from grapevine cultivars and Vitis species. Plant Molecular Biology Reporter 12:6–13.

Sallinen, S., A. Norberg, H. Susi, and A. L. Laine. 2020. Intraspecific host variation plays a key role in virus community assembly. Nature Communications 11:1–11.

Susi, H., D. Filloux, M. J. Frilander, P. Roumagnac, and A. L. Laine. 2019. Diverse and variable virus communities in wild plant populations revealed by metagenomic tools. PeerJ 2019.

Susi, H., A.-L. Laine, D. Filloux, S. Kraberger, K. Farkas, P. Bernardo, M. J. Frilander, D. P. Martin, A. Varsani, and P. Roumagnac. 2017. Genome sequences of a capulavirus infecting Plantago lanceolata in the Åland archipelago of Finland. Archives of Virology.
